# Prediction and Reduction of the Aggregation of Monoclonal Antibodies

**DOI:** 10.1016/j.jmb.2017.03.014

**Published:** 2017-04-21

**Authors:** Rob van der Kant, Anne R. Karow-Zwick, Joost Van Durme, Michaela Blech, Rodrigo Gallardo, Daniel Seeliger, Kerstin Aßfalg, Pieter Baatsen, Griet Compernolle, Ann Gils, Joey M. Studts, Patrick Schulz, Patrick Garidel, Joost Schymkowitz, Frederic Rousseau

**Affiliations:** 1 VIB Switch Laboratory, Herestraat 49, B-3000 Leuven, Belgium; 2 Department of Cellular and Molecular Medicine, KU Leuven, Herestraat 49, PO 802, B-3000 Leuven, Belgium; 3 Boehringer Ingelheim Pharma GmbH & Co. KG, 88400, Biberach/Riss, Germany; 4 EM-platform VIB Bio Imaging Core, VIB-KU Leuven, Herestraat 49, B-3000 Leuven; 5 Department of Pharmaceutical and Pharmacological Sciences, Laboratory for Therapeutic and Diagnostic Antibodies, KU Leuven, Herestraat 49, PO 820, B-3000 Leuven, Belgium

**Keywords:** APR, aggregation-prone region, FR, framework region, CDR, complementarity-determining region, RALS, right angle light scattering, FDA, US Food and Drug Administration, PDB, Protein Data Bank, MASS, mutant aggregation and stability spectrum, SEC, size-exclusion chromatography, SPR, surface plasmon resonance, protein folding, monoclonal antibody, protein aggregation, protein engineering

## Abstract

Protein aggregation remains a major area of focus in the production of monoclonal antibodies. Improving the intrinsic properties of antibodies can improve manufacturability, attrition rates, safety, formulation, titers, immunogenicity, and solubility. Here, we explore the potential of predicting and reducing the aggregation propensity of monoclonal antibodies, based on the identification of aggregation-prone regions and their contribution to the thermodynamic stability of the protein. Although aggregation-prone regions are thought to occur in the antigen binding region to drive hydrophobic binding with antigen, we were able to rationally design variants that display a marked decrease in aggregation propensity while retaining antigen binding through the introduction of artificial aggregation gatekeeper residues. The reduction in aggregation propensity was accompanied by an increase in expression titer, showing that reducing protein aggregation is beneficial throughout the development process. The data presented show that this approach can significantly reduce liabilities in novel therapeutic antibodies and proteins, leading to a more efficient path to clinical studies.

## Highlights

• Antibody aggregation continues to challenge the development of monoclonal antibodies for therapeutic applications.
• In the current manuscript, we apply our understanding of the interrelatedness of protein structure and aggregation and the particular role of aggregation gatekeeper residues to predict and engineer the solubility of monoclonal antibodies.
• This represents a novel rational approach to predict and redesign the solubility of monoclonal antibodies, an approach that could represent a significant improvement in the quality and safety of candidate selection during therapeutic antibody development.

## Graphical Abstract

**Figure:**
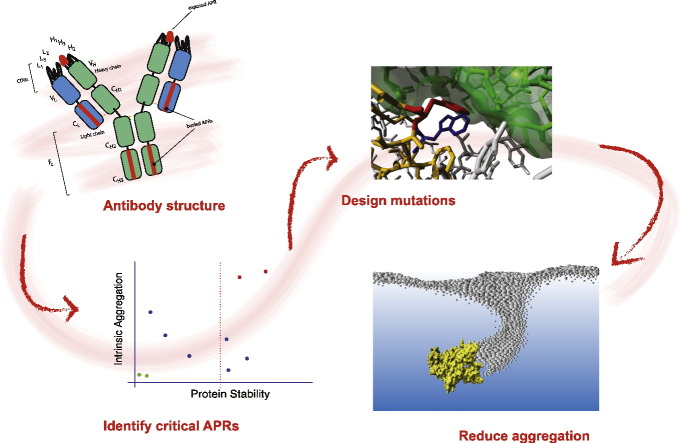

## Introduction

**Fig. 1:**
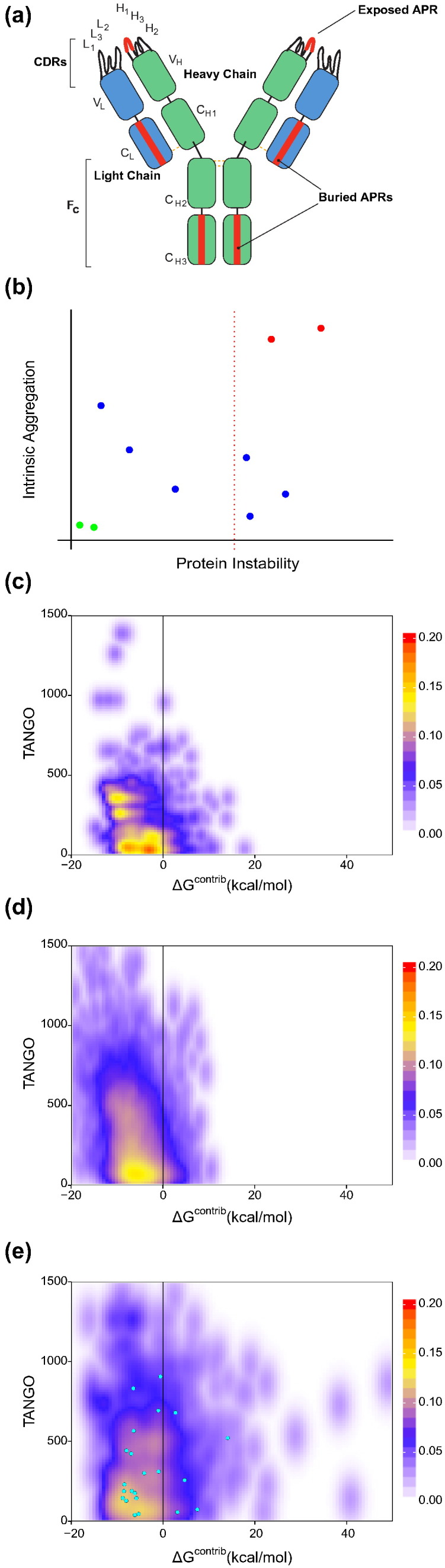
*In silico* analysis of aggregation propensity in antibody crystal structures. (a) Schematic representation of possible locations of APRs in monoclonal antibodies. APRs in CDRs would be more problematic than APRs buried in the immunoglobulin fold. (b) Stretch-plots: representation of aggregation propensity and local stability of APRs. Problems increase toward the top right of the plot; ideally, APRs would be located in the bottom left. (c) Density plot of all APRs located in the FR of over 2000 antibody structures from the abYsis database [24]. (d) Density plot of aggregation propensity and local stability of APRs in globular protein structures. The analysis is based on a set of 2650 high quality structures (*R*-factor of < 0.20 and resolution of < 1.9, with 30% sequence identity) of globular proteins generated using the Whatif software suite [25]. (e) Density plot of all APRs overlapping with CDRs of all antibody structure from the abYsis database. Cyan dots: APRs overlapping with CDRs of the 11 model antibodies used in the study.

Protein particles are assemblies built up of native and/or denatured proteins [1] that generally have a negative impact on manufacturability, stability, safety, titers, immunogenicity, and solubility of biologics in general [2], [3], [4], [5], [6]. Here, we investigate the impact of β-aggregation-prone sequences on protein particle formation and assess our ability to predict and suppress antibody particle formation based on this structural mechanism alone. β-aggregation is the process of association of proteins, predominantly through the formation of intermolecular beta-sheet structures by short aggregation-prone regions (APRs) of the polypeptide sequence [7]. Despite the fact that APRs are mostly hydrophobic in nature, they require other key properties such as a high β-sheet propensity and a low net charge. Common methods of aggregation prediction are geared toward the identification of APRs in the primary sequence [2], [3], [4], [5], [8], [9]. These prediction methods establish the theoretical aggregation potential of the protein in the unfolded state, called the “intrinsic aggregation propensity”. To nucleate aggregation, an APR must be solvent exposed in order to form stable interactions with other like sequences. However, in most globular proteins, these APRs are buried inside the hydrophobic core of the native structure, where they are prevented from triggering aggregation by the thermodynamic stability of the protein [7], [10], [11]. Noteworthy exceptions are APRs occurring at exposed sites of functional importance such as protein–protein interaction interfaces [12], [13], [14], [15]: here, the functional requirements of the site appear to lead to the emergence of APRs that can be problematic when the protein is not engaged in functional interactions. The effective aggregation of a protein is thus dependent on the population of aggregation-compatible conformations in which the APRs are exposed. The interplay of physicochemical parameters such as protein and ion concentrations, pH, and temperature contributes to determine the concentration of aggregation-prone conformers in a protein solution. Therefore, the challenge for relatively large and thermodynamically stable proteins like antibodies is identifying sequences that will aggregate under native conditions. The solution to this challenge lies in the distinction between APRs that are thermodynamically protected by folding and those that occur in aggregation-competent conformations that can form without major unfolding transitions (Fig. 1a). The latter regions would be the critical APRs that determine aggregation propensity under native conditions. We previously demonstrated for protective antigen and alpha-galactosidase that mutations in these critical APRs dramatically reduce the overall aggregation rate of the protein and increase the amount of soluble produced protein in mammalian cells. These mutations introduced the so-called suppressing gatekeeper residues that oppress aggregation locally [16]. Here, we investigated if the approach is transferable to the engineering of monoclonal antibodies, which is in itself non-trivial given the difference in architecture and size between antibodies and the previously studied cases. Moreover, we also wanted to test if the method would allow the sorting of aggregation-prone antibodies from less aggregation-prone ones, which would be extremely valuable in prioritizing lead candidates for therapeutic applications early during development.

Our publicly available web-based tool† [17] assumes that the distinction between a structural and a critical APR is largely determined by the local thermodynamic stability of the region containing the APR. A structural APR contributes to protein stability and is hence only available to trigger aggregation upon denaturation, whereas a critical APR can trigger aggregation under native conditions due to local structural fluctuations. To determine thermodynamic stability, we used here the empirical force field FoldX [18]. FoldX estimates the free energy of folding of protein structure by combining the local contributions of all residues, and thus, it is well suited to evaluate local stability (here called Δ*G*^contrib^, in kcal/mol). To detect the presence of APRs, we used the statistical thermodynamic algorithm TANGO [19]. TANGO calculates the intrinsic aggregation propensity of APRs as a Boltzmann distribution with competing secondary structural tendencies such as α-helical or β-hairpin structure. The benefit of this implementation is that TANGO predicts APRs [20] with well-defined sequence boundaries, that is, with a clear-cut separation between the APR and the surrounding residues. In addition, and of particular interest for our current purpose of identifying critical APRs, TANGO achieves high specificity and thus predicts few false-positive APRs [8]. Several other software packages were developed to help find aggregation hotspots in 3D structures [21], [22], [23]. As none of these methods consider both intrinsic β-aggregation potential and explicit all-atom thermodynamic stability calculations simultaneously, a force field such as FoldX, or an alternative, needs to be combined with an APR prediction method to obtain a complete method.

## Results

### Visualization

To easily visualize the aggregation potential of an antibody in function of both its intrinsic aggregation propensity and thermodynamic stability, we developed the so-called “Stretch-plot” (Fig. 1b). In a Stretch-plot, each APR is represented by a single point, of which the ordinate is determined by the local aggregation propensity of the sequence segment (summed TANGO, 5–100 per residue) and the abscissa by its contribution to the free energy of folding (Δ*G*^contrib^). In theory, APRs in the top right corner are “critical” since they occur in less stable regions of the protein and display higher intrinsic aggregation propensities. To analyze the relationship between intrinsic aggregation propensity and the local thermodynamic stability of antibody structures, we analyzed over 2000 antibody structures from the abYsis database [24]. For each of these antibodies, we performed a stretch-plot analysis by determining their APRs and their contribution to local thermodynamic stability. Finally, we combined the stretch-plots of all 2104 antibodies as heat maps (Fig. 1c and e). The analysis clearly revealed that APRs in the framework region (FR) are, in large majority, thermodynamically stable and therefore well protected from aggregation (Fig. 1c). In fact, the stretch-plot distribution of antibody FRs is very similar to the stretch-plot distribution obtained for a representative collection of globular proteins with various structural topologies (Fig. 1d, based on a representative set of high quality globular protein structures [25]). This similarity is in stark contrast to the stretch-plot distribution obtained for the complementarity-determining regions (CDRs) of the > 2000 abYsis antibody structures (Fig. 1e). APRs within CDRs display a much broader distribution of thermodynamic protection, and a significant proportion of these possess both high intrinsic aggregation propensity (TANGO) and low thermodynamic stability (FoldX), which would render them aggregation competent under near-native conditions. To investigate whether the results were susceptible to redundancy, we filtered with different sequence identity cutoffs, which did not have a profound effect on the overall distribution of the plots (Supplementary Fig. 1). The stretch-plot distribution of FRs is consistent with the notion that these sequences have been evolutionarily selected to minimize the occurrence of critical APRs rather than contain strongly stabilizing APRs in the hydrophobic core regions of their structure. On the other hand, CDRs are selected ad hoc for antigen binding and clearly explore more critical regions of the stretch-plot. The stretch-plot distribution of CDRs therefore indicates the emergence of potentially problematic APRs in CDRs as a result of paratope selection [3]. Indeed, analyses of antibody–antigen complexes have confirmed that aromatic residues (Tyr, Trp, and Phe), which are substantially enriched in APRs, are also considerably enriched in antibody paratopes [26], [27]. Interestingly, this is seen throughout protein–protein interaction sites [28], independent of fold or family. This raises the question as to what extent sequences can be selected to perform specific high affinity binding without facilitating the formation of associated APRs. The overlap between the physicochemical parameters determining the paratopes and those determining the APRs also underlines the importance of highly specific APR predictions to avoid the exclusion of false-positive “critical” antibodies. As aggregation is a concentration-dependent process, critical APRs are expected to be most challenging under conditions of high protein concentration, which occurs not only in therapeutic formulations but also, for example, in producer cells during recombinant expression.

### Test set composition and scoring function

**Fig. 2:**
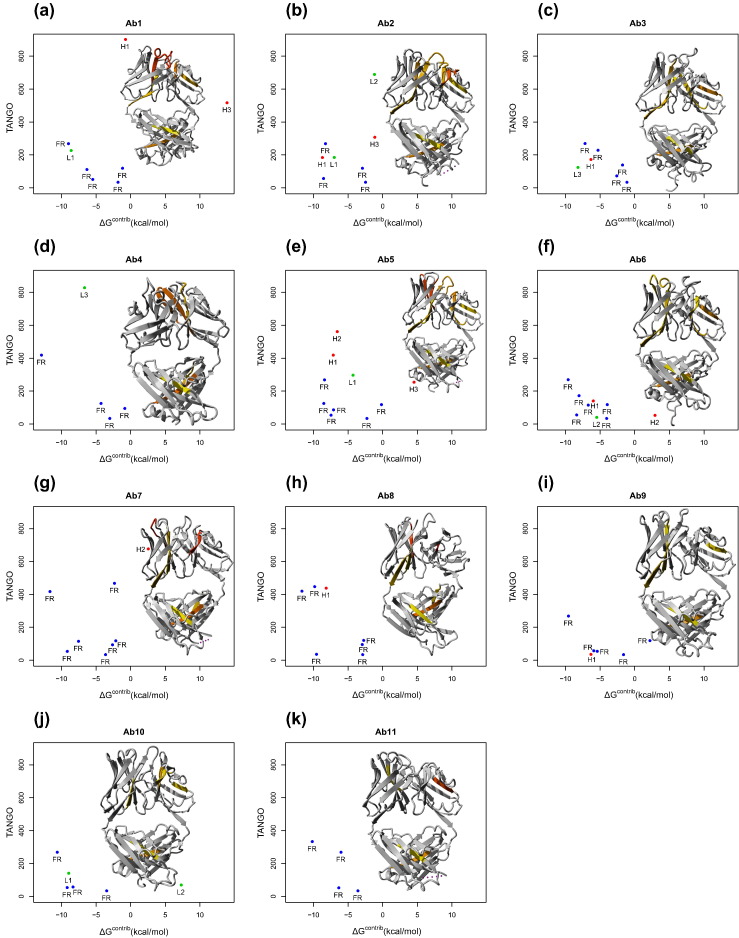
Stretch-plots and schematic representation of the structure of the Fab fragment of the 11 model antibodies used in this study. (a–k) Blue: APRs located in FR of the antibody. Red and green: APRs overlapping with CDRs in the heavy chain (H) or the light chain (L), respectively. Numbers represent CDR number (Chothia numbering) with which the respective APR is overlapping. Colors in structures: yellow: low scoring APR, red: high scoring APR.

To evaluate the impact of critical CDR-related APRs on the antibody's solubility and aggregation propensity, we decided to study the *in vitro* aggregation of a set of 11 publicly known antibody sequences (mAb1 to mAb11; Table 1). To compose this set, all available human sequences from the abYsis database were acquired and properties like charge distribution, hydrophobicity, statistical sequence scores [29], CDR-specific properties, and aggregation tendency (PASTA [30]) were computed. For each of these properties, a distribution was obtained and antibodies were selected that were extreme in at least one property and had an X-ray structure or a close homology modeling template available. Exceptions were mAb5, which scored average in all distributions, and mAb9 and mAb10 that were randomly selected for not having an X-ray structure available at that time. The selected antibodies contained APRs in their CDRs that span the majority of the data in the density plot analysis (Fig. 1e, cyan points correspond to the individual CDR APRs from the test cases). Moreover, the antibodies displayed a high diversity of stretch-plot profiles, suggesting that some are more aggregation-prone than others (Fig. 2). Four of the antibodies show APRs in their CDRs in the problematic region of the plot, namely mAbs 1, 2, 5, and 7. Interestingly, mAb9 does not conform to the general trend of Fig. 1 and has an APR in its FR of low thermodynamic stability. The other plots (mAbs 3, 4, 6, 8, 10, and 11) show no obvious problems.

**Fig. 3:**
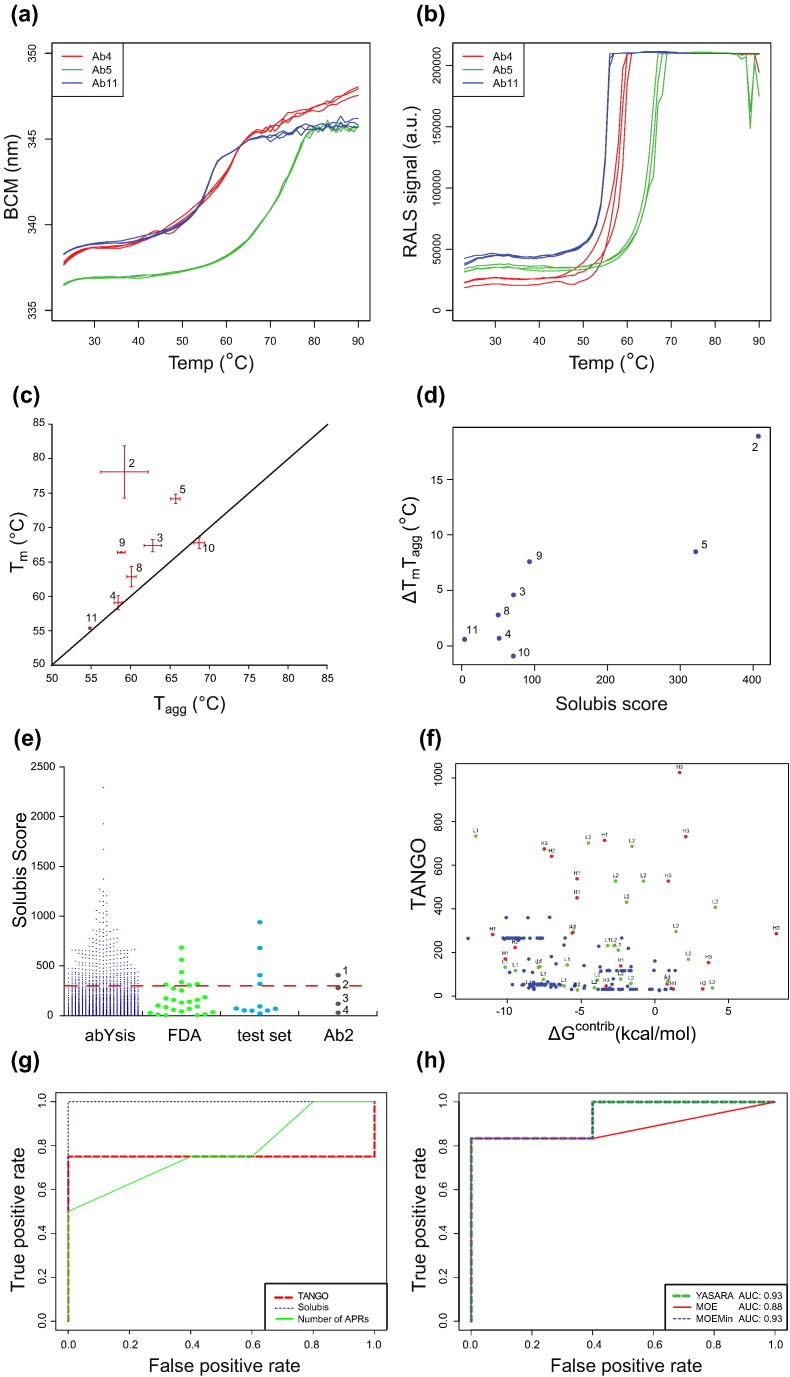
Characterization and scoring of the 11 model antibodies. (a) Temperature-dependent evolution of the barycentric mean (BCM) of the fluorescence emission spectrum of three representative antibodies from our test set. Curves were used to derive the melting temperatures (*T*_m_). (b) Temperature-dependent evolution of the RALS intensity measured simultaneously with the data in (a). The aggregation onset temperature *T*_agg_ is derived from these data. (c) A plot of the melting points and aggregation onset temperatures of all the test antibodies obtained from stably transfected CHO DG44 cells measured at 0.7 mg/mL. mAb numbers are indicated. (d) Correlation between the Solubis score and the difference between the *T*_m_ and the *T*_agg_ (Δ*T*_m_*T*_agg_) for the tested antibodies. (Pearson's correlation = 0.89, *p* < 0.02). mAb numbers are indicated. (e) Distribution of Solubis score over the different datasets. 1: WT, 2: FH101P, 3: SL50K, 4: SL50K_FH101P. (f) Stretch-plot summarizing the aggregation propensity and local stability of all APRs identified using TANGO in 27 FDA-approved monoclonal antibodies. Blue: APRs located in the FR of the antibody. Red and green: APRs overlapping with CDRs in the heavy chain (H) or the light chain (L), respectively. Numbers represent CDR number (Chothia numbering) with which the respective APR is overlapping. (g) Receiver operator curve showing the ability of the Solubis scoring function, TANGO, and the number of APRs to classify the WT antibodies structures, based on original crystal structure or homology model built using one template (Table 1). (h) Receiver operator curve similar to (g) but calculated using the results from different homology modeling approaches.

To extract critical APRs from the stretch-plot of each antibody and represent these by a single metric, we developed a simple integrative scoring function. A corrected TANGO score for each APR was devised by cutting off the Δ*G*^contrib^ at − 5 and + 5 kcal/mol and by normalizing this value between 0 and 1 (0 being a thermodynamically stable APR, 1 being an unstable APR). Multiplying this value with the TANGO score allows the mitigation of stable APRs in a way that the total score is dominated by the contribution of critical APRs. We named this scoring scheme “Solubis score” and set out to compare it to experimental data obtained using our set of 11 antibodies. We were not able to produce mAb1 from stably transfected CHO DG44 cells, and mAb6 was found to fragment. We experimentally determined thermal protein unfolding and aggregation of all remaining antibodies. Protein unfolding was monitored by intrinsic fluorescence (Fig. 3a) while simultaneously detecting aggregation using right angle light scattering (RALS; Fig. 3b) [31], [32], yielding melting (*T*_m_) and aggregation onset (*T*_agg_) temperatures, respectively. Substantial aggregation of mAb7 prior to the analysis prevented us from performing this analysis for mAb7. A plot of *T*_m_ *versus T*_agg_ for the remaining antibodies (Fig. 3c) shows that some of the tested antibodies aggregated from the native conformation, that is, prior to the global unfolding transition (*T*_agg_ < *T*_m_), while for other antibodies, aggregation onset only occurs upon global unfolding (*T*_agg_ = *T*_m_). This leads to the suggestion that their exposed APRs play a major role in aggregation. Importantly, no antibody resists aggregation past the global unfolding transition, consistent with the exposure of the APRs that are found in the stable regions of all antibodies of the set (the antibody with the lowest total number of APRs in this set has four). As mentioned above, the stretch-plot analysis in Fig. 2 predicted antibodies 1, 2, 5, 7, and 9 to have APRs in unstable regions of the structure, that is, to be aggregation-prone under native conditions. Of these, mAb1 could not be produced; mAb7 aggregated prior to any analysis; and mAbs 2, 5, and 9 displayed the largest difference between the *T*_m_ and *T*_agg_ values (18.9 °C, 8.5 °C, and 7.6 °C, respectively). In contrast, among the antibodies with no obvious problematic APRs, mAb6 displayed fragmentation during production and had to be discarded, and mAbs 4, 11, and 10 showed a difference between *T*_m_ and *T*_agg_ of less than 1 degree, whereas antibodies 3 and 8 had intermediate values (4.6 °C and 2.8 °C, respectively). Based on these observations, there seems to be a clear trend toward earlier aggregation onset for the antibodies with critical APRs in their stretch-plots. This can also be seen from the plot of Solubis score *versus* the difference between *T*_m_ and *T*_agg_, again showing that low-scoring antibodies tend to have small *T*_m_‐*T*_agg_ differences (Fig. 3d). Although the size of our current dataset precludes deriving firm conclusions on the general applicability of the scoring method, these results do suggest that the method allows the identification of problematic sequences that can drive antibody aggregation under native conditions. In summary, TANGO identified between 4 and 10 APRs in these antibodies, which Solubis reduces to 0, 1, or 2 APRs that are critical for native state aggregation.

For this reason, we compared the Solubis scores of the abYsis database to those of the antibodies currently approved for human therapy by the US Food and Drug Administration (FDA), for which we could find sufficient structural data (27 sequences; Fig. 3e and f). The plot depicts a Solubis score ranging from 0 to 2500 for the abYsis database, compared to a range of 0 to 685 for the FDA-approved sequences. Less than 15% of the FDA-approved sequences score above 300, a score that in our dataset was clearly associated with aggregation. The fact that FDA-approved antibodies are, according to our scoring, not completely devoid of exposed aggregation-prone sequences could suggest that our scoring is too stringent. However, the FDA-approved antibodies are formulated such that product stability is optimized and aggregation minimized, while our test set of nine mAbs was prepared in aqueous solution without any stabilizing agents. The fact that moderately aggregation-prone antibodies can meet safety and efficacy requirements indicates that the selection of antibodies can be especially improved toward favorable chemistry, manufacturing, and control properties.

### Robustness

Structure-based scoring functions are dependent on the quality and accuracy of a crystal structure or a suitable homology model. During early development, crystal structures are unavailable in most of the cases. However, a reliable homology model can also serve as a structural basis. Structure quality and accuracy are a prerequisite, since the scoring relies on the calculation of Δ*G*^contrib^ by FoldX. As this value is used to correct the raw TANGO score, it thus has a profound effect on the Solubis score. Despite the fact that there is a high degree of structural conservation in the antibody scaffold, a particular problem with explicit all-atom force field calculations is that they are very sensitive to relatively small errors in atomic coordinates. Thus, in order to assess the impact of the modeling algorithm on the scoring of the test set, we wanted to analyze the impact of different methods to build the homology models on our predictions. Hence, we used the software programs YASARA [35] and MOE [36]. For MOE, we prepared model structures with and without an additional energy minimization step.

To allow for easy comparison between these different models, we required a method to assess the performance of our scoring function in classifying aggregation-prone from soluble antibodies. To this end, we discarded mAbs 1 and 6 from the analysis and divided the remaining sequences into low and high aggregation-prone. Since the size of our dataset is too small to unequivocally resolve the twilight zone, we arbitrarily placed the cutoff in *T*_m_‐*T*_agg_ at 5 °C and included mAb7 in the high aggregation-prone class. Although this is obviously flattering for our approach, this classification does allow us to easily detect a negative impact of modeling methods on the prediction success. Prediction performance of a classification task can be visualized by a receiver operator curve, which plots for each cutoff value of our Solubis scoring function for critical APRs the fraction of correct predictions *versus* the fraction of false-positive predictions (Fig. 3g). A random scoring function will typically show a wrong prediction for every correct one made, and hence, its trace will lie near the diagonal. In contrast, the Solubis score calculated from the original structures lies on the curve through the upper left hand corner of the plot, which means that the function can flag the aggregation-prone antibodies in this small set with the highest sensitivity (zero false-negative rate) and high specificity (zero false-positive rate). In this set, where we know which antibody is aggregation-prone and classified it as such, the Solubis score clearly outperforms simpler scoring schemes, such as the total raw TANGO score or the number of APRs identified by TANGO in the sequence. For completeness, the Matthews correlation coefficient was 0.83, and the area under curve was 0.93 for the YASARA models and the MOE models with an additional energy minimization step. Although there are some differences between the scores obtained with each method, the overall scoring remains very similar for all antibodies with minimal impact on the prediction performance, which is apparent from comparing the receiver operator curves (Fig. 3h) of the different modeling methods. This result shows that the accuracy of state-of-the-art modeling engines allows the identification of critical APRs with similar confidence.

### Reducing aggregation by mutational APR suppression

**Fig. 4:**
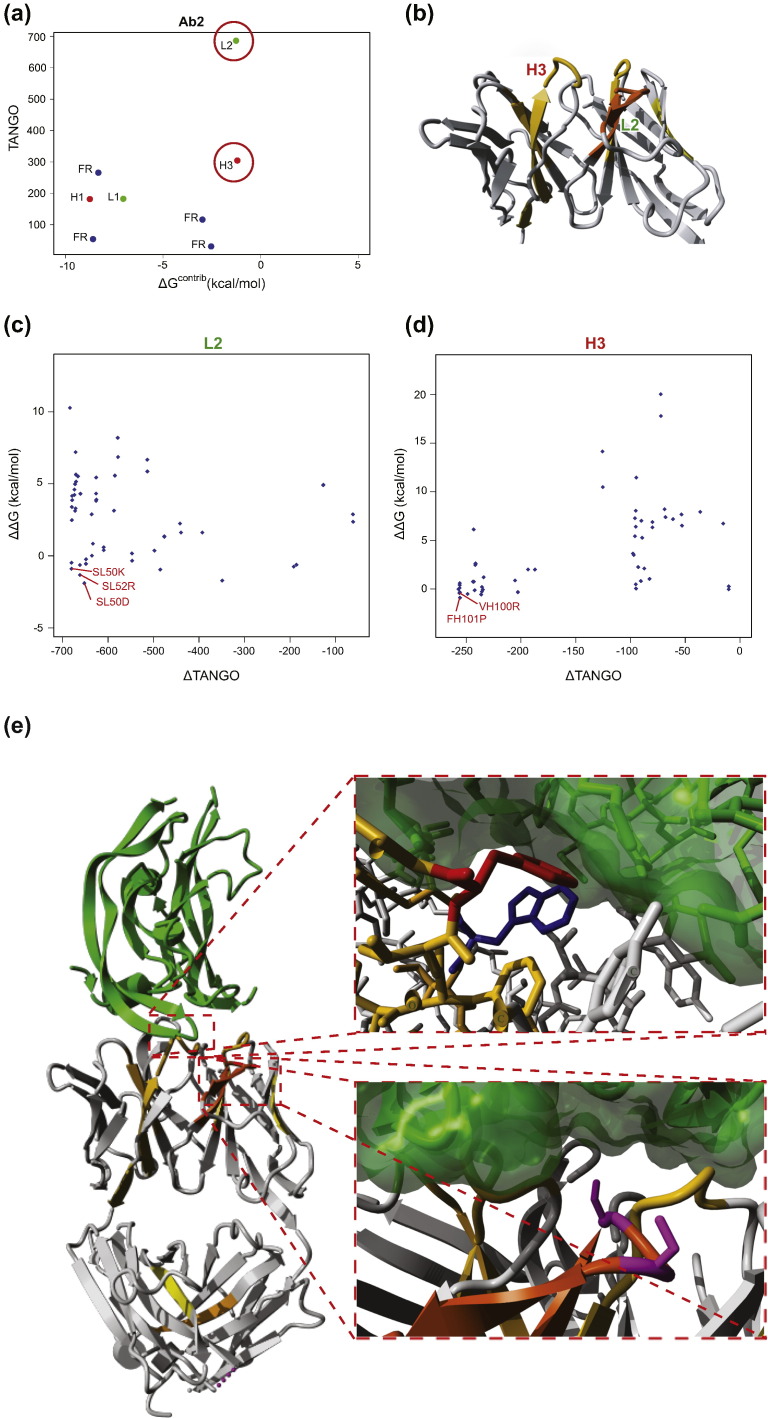
Design of APR disrupting mutations. (a) Stretch-plot of mAb2; critical APRs are highlighted with red circles. (b) Crystal structure of the variable domain of mAb2 with the APRs highlighted. (c–d) MASS plots of the two critical APRs in mAb2 with mutation effects on aggregation propensity and stability. Chosen mutations are highlighted in red. (e) Left: Crystal structure of mAb2 in complex with VEGF. Gray: mAb2, green: VEGF. Top right: Zoom on the APR in the heavy chain. Red: FH101, blue: Tryptophan located close to FH101, green: VEGF with molecular surface displayed. Bottom right: Zoom on the APR in the light chain. Magenta: SL50 and SL52, green: VEGF with molecular surface displayed. Images were made using YASARA Structure.

To further demonstrate the relevance of critical APRs, we decided to employ the same computational approach to select mutations that are predicted to suppress APRs and to study their effect on antibody production and aggregation. For this purpose, we focused on mAb2 {Protein Data Bank (PDB) ID 2FJF [33]}, which has a Solubis score of 407 and displays pronounced protein aggregation under native conditions, but the protein can still be expressed and purified with reasonable yield. Indeed, the low *T*_agg_ and large *T*_agg_‐*T*_m_ gap of mAb2 (Fig. 3c) indicates this antibody is particularly prone to aggregation from the native state. Previous work showed that a decrease of aggregation propensity can be achieved by the introduction of specific residues that oppose aggregation, called aggregation gatekeepers [10], [16], [34]. These residues are either charged residues (Aspartate, Glutamate, Arginine, or Lysine) or a Proline residue, which strongly disfavors a β-strand conformation. The stretch-plot of mAb2 reveals the identity of two critical APRs located in CDR L2 and H3, respectively (Figs. 2b and 4a and b). To identify mutations that reduce the intrinsic aggregation propensity of these APRs while not decreasing their contribution to the thermodynamic stability of the antibody, we calculated the effect on the aggregation propensity and protein stability of every mutation of APR residues to a gatekeeper (i.e., five mutations per position in the APR) and generated the so-called mutant aggregation and stability spectrum (MASS) plots [35] (Fig. 4c and d). In a MASS plot, each mutation is represented as a point of which the ordinate is determined by the change in intrinsic aggregation propensity (TANGO) associated with the mutation and the abscissa by ΔΔ*G*, that is, the change in thermodynamic stability (FoldX). Using this approach, we identified and selected mutations that were predicted to maximally reduce the aggregation propensity of the critical APRs in the CDRs of mAb2 with the minimal unfavorable effect on the stability of the protein. From the 55 possible mutations in L2, we selected SL50K, SL52R, and SL50D for further evaluation, and from the 65 calculated mutations in H3, we picked FH101P and VH100R (Fig. 4c, d, and e). These mutations were predicted to render the APR less aggregation-prone while not destabilizing the protein too much, both locally and globally.

### Improving colloidal stability through net charge increases

**Fig. 5:**
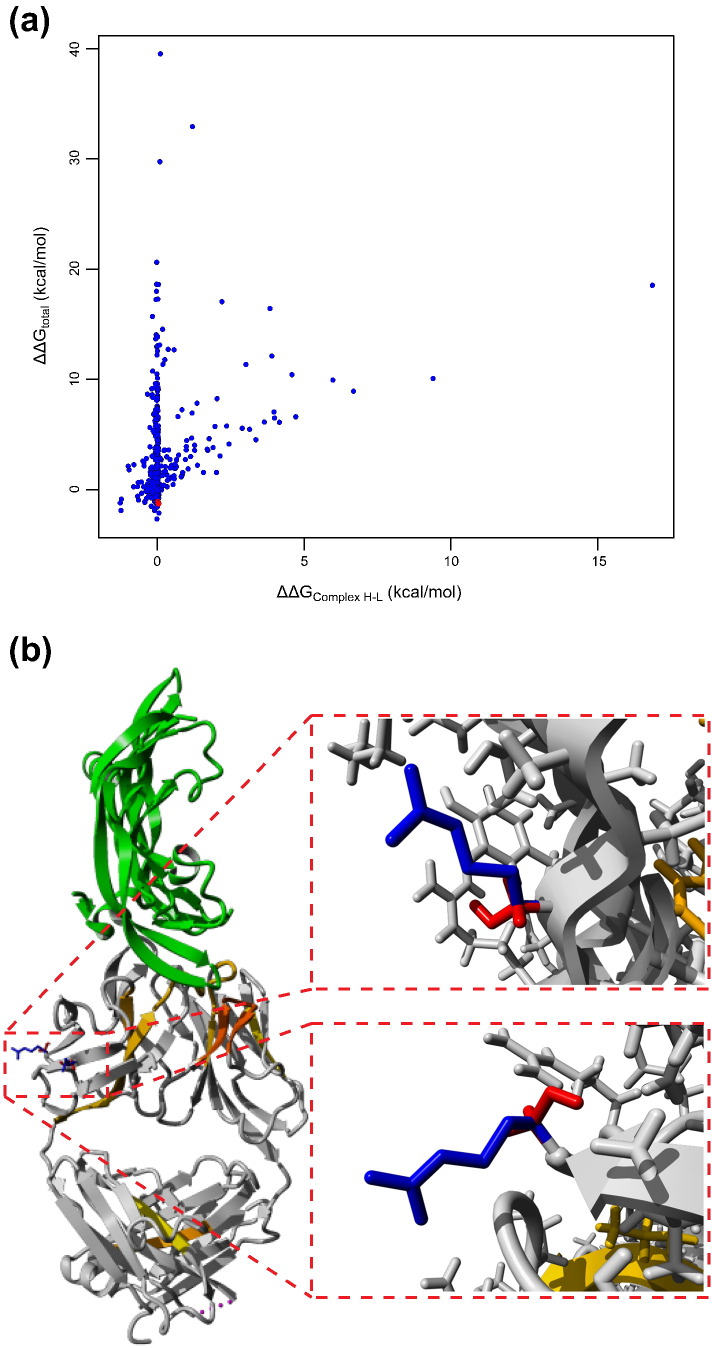
Design of net charge increasing mutation in the heavy chain. (a) Effect of all mutations on the global stability (Δ*G*) and stability of the complex between the heavy and light chain. Blue: Mutations that were not selected. Red: Selected mutations. (b) Left: Crystal structure of mAb2 in complex with VEGF. Gray: mAb2, green: VEGF. Top right: Zoom on the net charge increasing mutation SH21R. Red: SH21, blue: RH21. Bottom right: Zoom on the net charge increasing mutation SH85R. Red: SH85, blue: RH85. Images were made using YASARA Structure.

Given the clear overlap between antigen binding determinants and aggregation, we decided to supplement the mutations that directly disrupt the critical APRs by introducing suppressing gatekeepers with mutations that act on the global net charge of the proteins. Although this would not eliminate the intrinsic aggregation potential of the molecules, it would at least increase their colloidal stability and hence reduce the initial association required to initiate protein aggregation [36]. In addition, APR disruption could display an interplay with the net charge, as was observed in the engineering of protective antigen [16], where we noticed that the gatekeeper that increased the net charge of the protein also performed best at reducing aggregation. Moreover, for green fluorescent protein, it was previously shown that extreme supercharging is effective at suppressing aggregation, but this is probably not an option for therapeutic molecules due to immunogenicity considerations [36]. As it is known that heavy chain CDR 3 is often critical for antigen binding, we figured that direct APR disruption would most likely impact binding affinity. Therefore, we set out to test alternative mutations that act by supercharging the heavy chain. To this end, we used FoldX to calculate the ΔΔ*G* value of each mutation that increased the net charge of the heavy chain outside the CDRs, which, in the case of the mAb2 heavy chain, is positive (+ 2). This yielded 627 potential mutations (Fig. 5a), from which we selected 2 additional mutations in the heavy chain, namely SH21R (ΔΔ*G* = − 1.2 kcal/mol) and SH85R (ΔΔ*G* = − 1.2 kcal/mol; Fig. 5a and b). These mutations were combined with the light chain APR disrupting mutations SL50K and SL50R, in order to see if the net charge increase could take the role of APR disruption in the heavy chain. Moreover, we also added the net charge mutations to the combination of heavy chain and light chain APR disruptors, giving rise to the set of mutations shown in Table 2.

### Effect of mutation on titer and aggregation

**Fig. 6:**
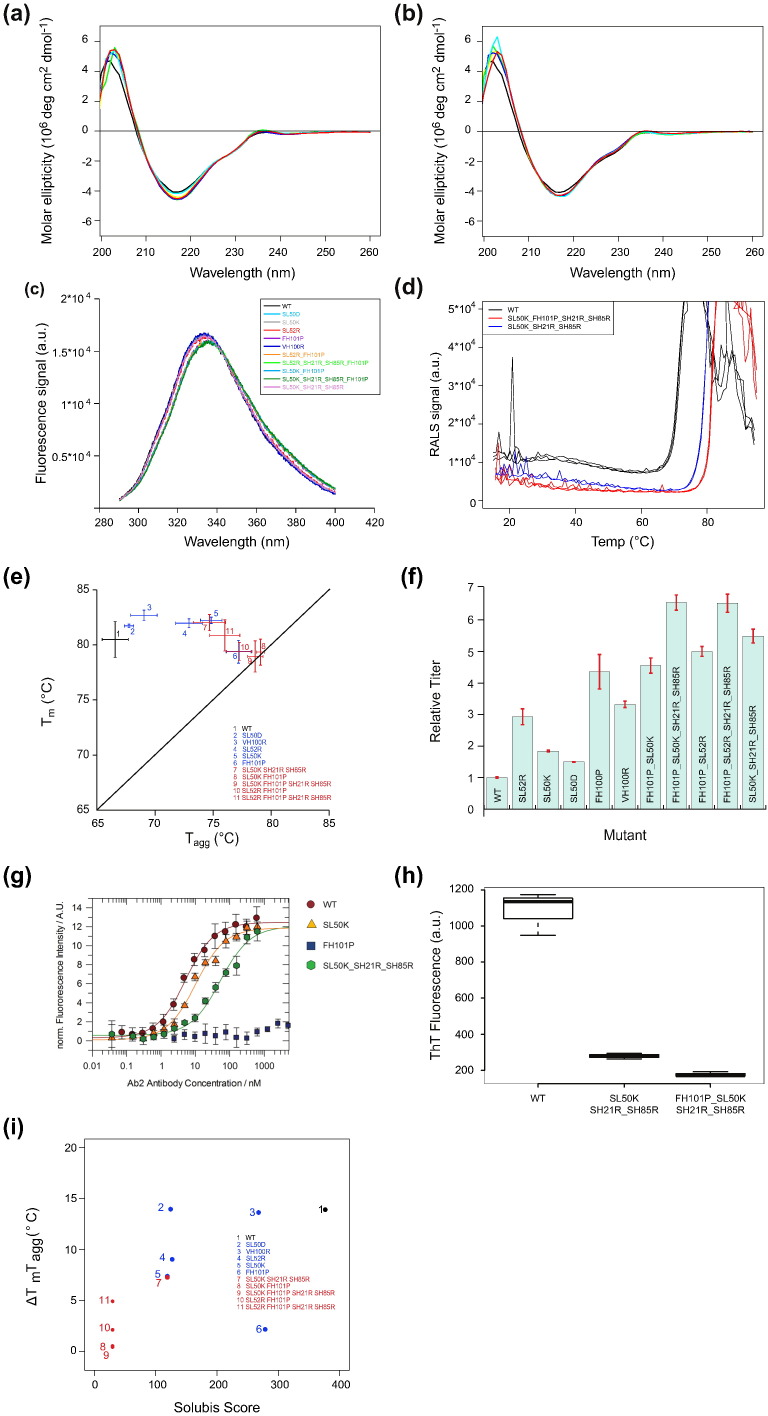
Characterization of mAb2 mutants. (a) Far-UV CD spectra. Black: Wild-type, yellow: SL50K, green: SL50D, blue: FH101P, cyan: VH100R, red: SL52R. (b) Far-UV CD Spectra. Black: Wild-type, yellow: SL52R_FH101P, green: SL52R_SH21R_SH85R_FH101P, blue: SL50K_FH101P, cyan: SL50K_SH21R_SH85R_FH101P, red: SL50K_SH21R_SH85R. (c) Intrinsic fluorescence emission spectra upon excitation at 20 °C of mAb2 and indicated mutants. (d) Temperature-dependent evolution of the RALS intensity for wild-type and two mutants. (e) Aggregation onset points and melting temperatures of wild-type and mutants at 1 mg/mL, obtained from transiently transfected CHO K1 cells. (f) Expression titers for mAb2 wild-type and mutants. (g) VEGF binding determination using optical laser-induced thermophoresis of mAb2 WT and selected mutants. (h) Fluorescence intensity of the rotor dye Thioflavin-T in the presence of mAb2 WT and the mutants SL50K_SH21R_SH85R and FH101P_SL50K_SH21R_SH85R. Excitation was at 440 nm, emission was recorded at 480 nm. (i) Correlation between the Solubis score and the difference between the *T*_m_ and the *T*_agg_ (Δ*T*_m_*T*_agg_) for mAb2 wild-type and mutant; numbers are indicated.

The mutants selected for the analysis (listed in Table 2) were recombinantly produced and purified from transiently transfected CHO K1 cells alongside wild-type mAb2. Comparison of the intrinsic fluorescence emission and CD spectra of the mutant and wild-type confirmed that the mutations did not cause major alterations in the overall structure of the antibody (Fig. 6a–c). In contrast, we observed major improvements in the aggregation onset temperature of the mutants (Fig. 6d). The plot of the *T*_m_ of the mutants *versus* their *T*_agg_ showed that several of the single mutations improved the aggregation onset temperature, without decreasing the thermodynamic stability of the protein (Fig. 6e). The strongest improvements of *T*_agg_ were obtained with APR suppressing mutations, where S50K in the light chain and FH101P in the heavy chain performed best, and their combination is the best mutant overall in this plot. The combination of these two mutations (variant 8 in Fig. 6e) completely eliminated APR-driven native state aggregation of mAb2. Variants 8 and 9 of mAb2 display a 15 °C shift of *T*_agg_, so that *T*_agg_ now equals *T*_m_, and their aggregation is only initiated upon the global unfolding of the antibody. The net charge increasing mutations did not cause much further improvement on top of the effect from the APR (see variants 7 and 9 in Fig. 6e). Interestingly, the reduction of aggregation propensity was associated with an increase in the expression titer of the same mutants (Fig. 6f). Variant 8 had an expression titer that was 4.5-fold higher than wild-type mAb2, which was 6-fold for variant 9. For both variants, the net charge increasing mutations had an additional effect in terms of expression titer. (Fig. 6f). The improved physicochemical properties of these Solubis-designed mutants were further corroborated from the reduced presence of aggregates in the protein stocks as confirmed by dynamic light scattering and transmission electron microscopy (Supplementary Figs. 2 and 3). Additionally, size-exclusion chromatography (SEC) showed an increase in monomer content, measured immediately after purification (Supplementary Fig. 4), all indicating that the improvements are substantial and detectable using orthogonal methods.

### Overlap with paratope binding requirements

These data demonstrate that critical APRs are a strong determinant of the aggregation and expression titer of antibodies, since the mutational suppression of such regions leads to significant improvements of both properties. However, the overlapping physicochemical requirements of paratopes and APRs, which are both enriched in hydrophobic/aromatic residues, will very likely result in a trade-off between ligand binding and aggregation propensity. In order to test the ability of mAb2 variants to still bind their ligand, we performed surface plasmon resonance (SPR) and microscale thermophoresis binding experiments of wild-type mAb2 and variants thereof with its native antigen human vascular epithelial growth factor (Fig. 6g and Table 2). We found that variants incorporating substitutions in the light chain of mAb2 can still bind human vascular epithelial growth factor with affinities comparable to wild-type. On the other hand, the best aggregation-reducing mutation in the APR of the heavy chain, FH101P, resulted in a complete loss of antigen binding, which is in line with the known importance of heavy chain CDR3 for antigen recognition [37]. Therefore, considering both ligand binding and aggregation propensity, the most successful mAb2 variant (Variant 7) has a Solubis score of 120, exhibits a *T*_agg_ improvement of 8 °C, and shows an expression titer increase of more than 500% over the wild-type. The favorable properties of the mAb2 SL50K_SH21R_SH85R variant were experimentally confirmed by the absence of aggregates in the stock solution (stored at 4 °C) as observed by transmission electron microscopy (Supplementary Fig. 3) and a reduced binding to the rotor dye Thioflavin-T (Fig. 6h). We also confirmed the improvement of the mAb2 SL50K_SH21R_SH85R variant with a long-term stability study over a time period of 180 days (Table 3) at 40 °C. The variant shows less aggregation at *t* = 0 and a slower decrease of monomer content overtime when compared to the wild-type.

## Discussion

In conclusion, we developed a scoring scheme that determines the risk for antibody aggregation under native conditions. Our algorithm considers not only the intrinsic aggregation propensity of the primary antibody sequences but also the structural context in which these aggregation-prone sequences are embedded. Thus, starting from the full set of APRs in an antibody's primary sequence, the scoring function identifies those that most likely actually trigger aggregation in the fully folded protein under native conditions. In our test set of 11 antibodies, the total number of APRs in the primary sequence of the different antibodies ranges between 4 and 10 APRs. However, only in a fully unfolded and extended state of a polypeptide will all these APRs be available for aggregation.

In antibodies, just as in any globular protein, most APRs will be buried in the hydrophobic core of the protein where they will contribute to thermodynamically stabilizing tertiary interactions of the protein. This also means that these APRs are thereby protected from aggregation by their engagement in native interactions. The more stable these interactions, the more efficient this protection. Our algorithm therefore filters APRs by their contribution to the thermodynamic stability of the native protein. In our test set of 11 antibodies, this filtering lowers the amount of relevant APRs to zero, one, or two APRs per antibody (i.e., critical APRs). Based on these critical APRs only, the scheme allows us to correctly classify the antibodies in this limited set into antibodies that are at risk of aggregating in their native conformation and antibodies that are only at risk of aggregation when they globally denature (e.g., melt upon heating). Of course, given the canonical structure of antibodies, critical APRs, in their vast majority, will be part of CDRs. To demonstrate that the critical APRs predicted by our method are indeed responsible for native state aggregation, we reengineered the aggregation-prone antibody 2, which resulted in mutants with reduced native aggregation without abrogating epitope binding. Interestingly, we found that these same mutations also improve the expression titer of the antibody up to more than 4.5-fold for the best variants. These results demonstrate the importance of β-aggregation for the chemistry, manufacturing, and control properties of monoclonal antibodies and the importance of identifying critical APRs within CDRs.

The overlap between epitope binding and the aggregation potential of antibodies is a critical point. Indeed, as discussed above, APRs occurring in constant regions of the protein are typically buried in the hydrophobic core of one of the immunoglobulin domains and therefore contribute little to native state aggregation propensity. Therefore, a simple proxy for our scoring scheme could be to use the TANGO score of the CDRs to evaluate the aggregation propensity of antibodies, and indeed, in our test set, this would work reasonably well: only mAb4 would be wrongly classified as aggregation-prone. This antibody has a strong APR in CDR loop 3 of the light chain, but since this loop is stably integrated into the fold of this antibody, the presence of the loop does not result in a strong aggregation propensity under native conditions. Thus, although a larger dataset would be required to determine which method is more accurate, our data suggest that consideration of the full structural context will be more specific.

One could also argue that the overall TANGO score, unmitigated by the structural context, is a simpler method than the Solubis score in classifying the antibodies. With this approach, the presence of critical APRs in problematic antibodies then simply adds up on top of the sum of identical APRs in the constant or highly similar framework sequences and thus tends to correlate with a higher overall TANGO score. In classifying aggregation-prone antibodies, the Solubis score clearly outperforms the overall TANGO score for the wild-type antibodies (Fig. 3g and Table 4). Also, it is interesting to notice that there is not much predictive power in the number of APRs in an antibody, where the number of critical APRs is a powerful method. When looking whether the Solubis score correlates with the *T*_m_‐*T*_agg_ gap for mAb2 wild-type and mutants, we can see a clear trend, but mutant FH101P has a larger effect on the *T*_m_‐*T*_agg_ gap than would be expected from the Solubis score (Fig. 6i and Table 4). Additionally, mutant SL50D has a substantial effect on the Solubis score but did not affect the *T*_m_‐*T*_agg_ gap. Interestingly, this is the one mutation that reduces the net charge compared to wild-type, suggesting that this factor might need to be incorporated into future improvements to our scoring scheme. Although our dataset is perhaps too small to identify the best scoring scheme, it is clear that the method of identifying critical APRs achieves the best prediction power on the current set and, at the same time, identifies the sites where the protein might benefit from engineering.

After analyzing over 2000 antibody structures, we find that a substantial fraction of these possess CDRs with critical aggregation propensity. Indeed, epitope binding imposes specific sequence requirements on paratopes such as a bias toward aromatic sequences, which will also favor aggregation. Contrary to APRs in the core of the immunoglobulin fold, APRs in CDRs are not selected for structure but for binding. As a result, these APRs are generally not significantly contributing to the thermodynamic stability of the protein and therefore promote aggregation even when the antibody is properly folded.

Comparing the aggregation propensity of FDA-approved antibodies with the abYsis set of over 2000 antibodies clearly shows that CDRs with extreme Solubis scores do not possess the requirements allowing commercial exploitation. Interestingly, about 15% of FDA-approved antibodies analyzed here still possess at least one critical APR. This indicates that antibody formulation is an efficient method to stabilize monoclonal antibodies that may act in part by helping shield the critical APRs from aggregation. However, our results also suggest that identifying critical APRs can further support candidate selection of antibodies in the context of quality by design and risk management in development.

## Materials and Methods

### Database retrieval

The abYsis database [24] was queried with the PDB as data source, all organisms, excluding sequences with warnings and excluding unclassified, unpaired, and unnumbered sequences. This resulted in 2561 antibody structures. Duplicates were removed and also antibodies that contained errors after downloading, resulting in a database of 2104 antibody structures. Redundancy was removed using the CD-HIT webserver using sequence identity cutoffs of 0.95 and 0.90 [42].

The WHATIF culled dataset with representative PDB structures of globular proteins was obtained from http://swift.cmbi.ru.nl/gv/select/. Dataset was culled at 30% sequence identity for structures with an *R*-factor of < 0.20 and a resolution of < 2.5 Å [25].

### Selection of 11 publicly available antibodies from the abYsis database

All human sequences available in the abYsis database were acquired, and properties like charge distribution, hydrophobicity, statistical sequence scores [29], CDR-specific properties, and aggregation tendency (PASTA [30]) were computed. For each of these properties, a distribution was obtained and antibodies were selected that were extreme in at least one property and had an X-ray structure available. Exceptions were mAb5, which scored average in all distributions, and mAb9 and mAb10, which were randomly selected and for which no X-ray structure was available.

### Homology modeling

Homology modeling was performed using the “Antibody Modeler” application of MOE 2015.10 (Chemical Computing Group Inc., Montreal, Canada). The application was run fully automated according to the procedures described in the manual. However, templates that correspond to the available crystal structure of the test mAb were excluded. Side-chain clashing energy cutoff was set to 1.5 kcal/mol, intermediates were refined with the “medium” setting, and final models were refined with the “fine” setting. GB/VI scoring was employed, and energy minimizations were performed with the Amber10:EHT (R-Field) force field.

In a further approach, an additional energy minimization step was performed subsequent to the homology modeling (force field: Amber10:EHT; R-Field 1:80; Cutoff (8.10) ; root mean square gradient of 0.1 kcal/mol/A^2^).

Homology modeling with YASARA was performed using the homology modeling macro supplied with YASARA Structure (hm_build.mcr). Standard settings were used, and only the FASTA sequence of the heterodimer of the Fv-domain of each mAb was provided.

### In silico analysis

The following method was used for each antibody structure analyzed. APRs were identified using TANGO [19] at default settings, and the location was defined using the Chothia canonical numbering scheme [38]. All structures were cleaned and prepared for analysis using YASARA Structure [39], after which they were repaired using the FoldX force field [18]. The SequenceDetail command was run with FoldX for all repaired structures. Python scripting was used to retrieve the summed Δ*G* of each APR from the SequenceDetail files. R-Studio [40] was used to make Stretch-plots using the plot function and density plots using the stat_density2d function, both from the ggplot2 package [46]. The summed Δ*G* value was cut off between − 5 and 5 kcal/mol. This value was scaled between 0 and 1, after which it was used to scale all TANGO scores with this value, giving APRs with summed Δ*G* values of − 5 and lower a score of 0. For each antibody, all APR scores were summed, resulting in the score defined by the scoring function.

### Protein production and purification

Variable regions of mAb candidates were synthesized and inserts cloned into expression vectors already containing constant regions of an IgG1 isotype. Heavy Chain (HC) and Light Chain (LC) were cloned into individual expression vectors and co-transfected.

Wild-type mAbs were produced in stable clones of CHO DG44 cells in a 10-day fed-batch culture in CD-OptiCHO with a 10% bolus feed on day 3 and day 6 using CD EfficientFeed C (Life Technologies) [41].

mAb2 variants were produced by transient expression in CHO K1 suspension adapted cells. The seeds were grown in an optimized, chemically defined, animal-component free, and serum-free media. Cells were transfected with a proprietary transfection agent. After transfection, cells were grown in an optimized media with proprietary recipe at 37 °C and 5% CO2 for 8 days.

The proteins were purified from cell culture supernatant via protein A affinity chromatography (MabSelect or rProtein A Sepharose, GE Healthcare). The purified mAbs were analyzed by reducing and non-reducing SDS-PAGE. Quantification of mAb aggregation/fragmentation was performed by SEC.

### Production mAb2 variants

Variable regions of mAb candidates were synthesized and inserts cloned into a pTT5 expression vector (CNRC-NRC) already containing constant regions of an IgG1 isotype. HC and LC were cloned into individual expression vectors. CHO 3E7 cells (CNRC-NRC [42], [43]) were cultivated in shake flasks using FreeStyle CHO medium (Life Techologies) + 2 mM L-Gln. HC and LC expression vectors were co-transfected in a 1:1 plasmid ratio into CHO 3E7 cells with a DNA-to-PEIpro (Polyplus) ratio of 1:6. Each mAb variant was transfected in triplicates. Subsequently, cells were cultured for 8 days in shake flasks on an orbital shaker (Infors) set to 120 rpm, with 5% CO2 and 32 °C, using FreeStyle CHO medium (Life Techologies) + 2 mM L-Gln without selection pressure. At 24 h post-transfection, cells were supplemented with HyClone Cell Boost 5 (Thermo Scientific). The concentration of secreted IgG in the harvested culture media was determined on day 8 post-transfection with an Octet QKe (ForteBio) using Protein A biosensors.

### Target binding determination using optical laser-induced thermophoresis

The method description and experimental performance was described previously [44], [45]. Briefly, recombinant human VEGFa (UniProt ID P15692; aa 27–191) was fluorescently labeled with Alexa-647. Target binding affinities were determined in solution using labeled recombinant human Vascular Endothelial Growth Factor a (rhVEGFa) at a constant concentration of 1 nM, whereas mAb2 WT or variants thereof were added in varying concentrations. All measurements were performed in triplicates at 295 K, 20% LED power, and 20% IR- laser power. Laser-on and -off times were adjusted to 30 s and 5 s, respectively. All experiments were carried out in 25 mM sodium citrate buffer at pH 6.0 containing 125 mM sodium chloride and 0.05% Tween 20. Bevacizumab (Avastin®, Genentech, Inc.) was used for control experiments. All measurements were conducted on a NanoTemper Monolith NT.115 instrument. Data were analyzed using the NanoTemper Analysis version 1.5.41 (NanoTemper Technologies GmbH).

### Determination of melting point (*T*_m_) and aggregation onset temperatures (*T*_agg_)

*T*_m_ and *T*_agg_ of all antibodies were determined by intrinsic tryptophan fluorescence using the OPTIM 1000 (Avacta Analytical Ltd., York, UK) instrument. All experiments were performed in triplicates of triplicates. The analysis of all antibodies was done at a concentration of 0.7 mg/mL in 25 mM Na-Citrate and 125 mM NaCl (pH 6.0). mAb2 WT and variants were analyzed at a concentration of 1 mg/mL in 25 mM Na-Citrate and 125 mM NaCl (pH 6.0). For all experiments, a temperature ramp was performed from 15 to 95 °C, with an increase of 0.3 °C/min. Exposure time was set to 2000 milliseconds. Melting temperatures and aggregation onset temperatures were determined with the instrument software.

### CD

A J-1500-150ST (Jasco) CD Spectrometer with a peltier temperature control system was used to collect CD spectra. All measurements were collected at 25 °C between 190 and 260 nm with a pitch of 1 nm at a scan speed of 50 nm min^–1^, a response time of 4 s, slit widths of 2 nm, and standard sensitivity.

### Long-term storage stability/SEC

An accelerated aggregation study of mAb2 WT and the variant mAb2_SL50K_SH21R_SH85R was performed. Samples of 5 mg/mL in 25 mM sodium citrate and 125 mM sodium chloride (pH 6.0) were incubated at 40 °C over a time span of 6 months (180 days). The unstressed samples (*t* = 0) were analyzed immediately after preparation. All 40 °C incubated samples were stored at − 70 °C and analyzed together at the end of the study. The amounts of aggregation and remaining monomer content were determined by SEC on an Alliance HPLC system (Waters, Milford, MA, USA) employing a TSKgel G3000 SWX column (Tosoh Bioscience LLC). The monomer loss overtime was calculated as slope (m) of the line defined by the monomer content of a sample at the initial and end time point of the study.

The mobile phase was 50 mM Tris/HCl and 150 mM sodium chloride buffer (pH 7.5) at a flow rate of 1 mL/min. Areas of peaks followed by 280 nm are integrated at each time point. All samples were measured in duplicates.

### Electron microscopy

We deposited 10 μL of the samples (1 mg/ml) on glow-discharged, carbon-coated 400 mesh copper grids for 1 min. Subsequently, the grids were washed briefly 5 × on drops of MilliQ filtered H2O and stained for 1 min with 1% filtered uranyl acetate. After blotting and drying, the grids were observed in a JEOL JEM1400 transmission electron microscope equipped with an Olympus SIS Quemesa 11 Mpxl camera at an acceleration voltage of 80 kV.

### SPR

The affinity of mAb2 WT and mutants was determined by SPR using a Biacore 3000 analytical system (GE Healthcare, Uppsala, Sweden). mVEGF was covalently coupled (890RU) to a CM5 sensor chip [using a concentration of 2 μg/mL mVEGF in acetate buffer of 10 mmol/L (pH 4.5)]. mAb2 variants (diluted in HBS-EP buffer to concentrations between 10 and 200 nM) were injected at a flow rate of 30 μL/min, followed by a dissociation. After each cycle, the sensor chip was regenerated using glycine [10 mmol/L (pH 1.5)]. The association and dissociation rate constants were determined using the BIAcore 3000 evaluation software (Langmuir binding, global fit).

## Figures and Tables

**Table 1:** Antibody test set

Antibody	Antigen	PDB^1^	TANGO	Δ*G* corrected TANGO	Net charge
mAb1	unknown	3kyk[52]	2314	941	2
mAb2	VEGF	2fjf[37]	1908	377	3
mAb3	HIV	1hzh[53]	1131	71	10
mAb4	MHCI	3hae[54]	1621	51	3
mAb5	EGFR	3b2u[55]	2269	321	3
mAb6	GP41	1tzg[56]	1064	53	9
mAb7	GP41	3mac[57]	2042	681	− 1
mAb8	HIV-UG29	2b0s[58]	1639	50	− 3
mAb9	DNA	1dfb[59]	680	93	5
mAb10	TNF	1ad9[60]	695	71	3
mAb11	CD4	2adg[61]	771	4	− 6

1 PDB IDs of crystal structure of the antibody itself or the YASARA homology modeling template (underlined).

**Table 2:** mAb2 mutations and their binding properties to VEGF

VEGF binder	Thermophoresis *K*_D_ (nM)	SPR *K*_D_ (nM)
mAb2_WT	3.7 ± 1.1	4.22
mAb2_SL50K	8.3 ± 1.4	7.88
mAb2_SL50D	4.4 ± 1.3	4.68
mAb2_SL52R	3.2 ± 1.8	2.86
mAb2_VH100R	*NB*	*NB*
mAb2_FH101P	*NB*	*NB*
mAb2_SL50K_SH21R_SH85R	57.1 ± 6.2	–
mAb2_SL50K_FH101P	*NB*	–
mAb2_SL50K_SH21R_SH85R_FH101P	*NB*	–
mAb2_SL52R_FH101P	*NB*	–
mAb2_SL52R_SH21R_SH85R_FH101P	*NB*	–
Bevacizumab (Avastin)	4.8 ± 1.6	~ 1.8–20 [33], [46]

*NB*: No binding under assay conditions used.

–: Data not gathered.

**Comment:** Bevacizumab was used for control experiments. The obtained binding affinity is in agreement with currently available published data (*K*_D_ of ~ 1.8–20 nM; measured by SPR) [33], [46].

**Table 3:** Monomer loss overtime for wild-type and mutant mAb2

	Monomer loss overtimem (per day)	Monomer portion average initial (*t* = 0)	Monomer portion average (*t* = 180 days) (% area)
mAb2_WT	− 0.0938	79.10	62.22
mAb2_SL50K SH21R SH85R	− 0.0179	97.02	93.80

The monomer loss overtime was calculated as slope (m) of the line defined by the monomer content of a sample at the initial and end time point of the study.

**Table 4:** Summary of antibodies used for scoring function validation

Antibody	TANGO	Solubis Score	Class	No. of APRs	Mutant
mAb2	1908	377	1	8	No
mAb3	1131	71	0	7	No
mAb4	1621	51	0	6	No
mAb5	2269	321	1	10	No
mAb7	2042	681	1	7	No
mAb8	1639	50	0	7	No
mAb9	680	93	1	5	No
mAb10	695	71	0	5	No
mAb11	771	4	0	3	No
mAb2 VH100R	1652	268	1	8	Yes
mAb2 FH101P	1653	279	0	8	Yes
mAb2 SL50D	1256	125	1	7	Yes
mAb2 SL50K	1228	119	1	7	Yes
mAb2 SL50K SH21R SH85R	1228	119	1	7	Yes
mAb2 SL52R	1246	127	1	7	Yes
mAb2 FH101P SL50K	972	30	0	7	Yes
mAb2 FH101P SL50K SH21R SH85R	972	30	0	7	Yes
mAb2 FH101P SL52R	991	30	0	7	Yes
mAb2 FH101P SL52R SH21R SH85R	990	30	0	7	Yes

## Appendix A Supplementary Data

Supplementary figures Image 2

## Footnotes

† http://solubis.switchlab.org/
