# Supplementary material for: Prediction and Reduction of the Aggregation of Monoclonal Antibodies
Source: J Mol Biol. 2017 Apr 21;429(8):1244–61. doi: 10.1016/j.jmb.2017.03.014 (PMC5397608; doi:10.1016/j.jmb.2017.03.014)
Supplement: Supplementary file 1 — Supplementary figures [file mmc1.docx]

Van der Kant *et al.*

**Prediction and reduction of the aggregation of monoclonal antibodies**

**Supplementary Materials**

**Supplementary figure 1.** Effect of redundancy removal on density plots. (a,b) No redundancy removal, containing info from 2104 antibody structures. (c,d) 95% redundancy removed. (e,f) 90% redundancy removal. (a, c, e) APRs in Framework Region. (b, d, f) APRs overlapping with CDRs. Number of APRs is listed above the respective plots.

**Supplementary figure 2.** Dynamic light scattering of stocks of Ab2 WT and mutants diluted to 1mg/mL. a) WT. b) SL50K. c) SL50D. d) FH101P. e) VH100R. f) SL52R. g) SL50K_SH21R_SH85R. h) SL50K_FH101P. i) SL50K_FH101P_SH21R_SH85R. j) SL52R_FH101P. k) SL52R_FH101P_SH21R_SH85R.

**Supplementary figure 3.** Transmission Electron microscopy images of Ab2 (A) and the mutant SL50K_SH21R_SH85R (B). Samples were diluted to 1mg/mL and stained with uranyl acetate.

**Supplementary figure 4.** Initial monomer content of purified samples of Ab2 and mutants assessed by Size Exclusion Chromatography. Aggregate Level Determination was performed by HP-SEC using a Tosoh TSK G3000 SWX resin with precolumn and by applying a flow of 1mL/min.
